# Can we determine Alzheimer’s disease neuropathology from the finger?

**DOI:** 10.1002/alz.092341

**Published:** 2025-01-09

**Authors:** Hanna Huber, Laia Montoliu‐Gaya, Silke Kern, Anna Dittrich, Barbara Borroni, Daniele Altomare, Millie Sander, Anne Corbett, Núria Aguilera, Raúl Núñez‐Llaves, Fernando García‐Gutiérrez, Andreas Jeromin, Richard S. Isaacson, Sebastian Palmqvist, Oskar Hansson, Erik Stomrud, Mercè Boada, Xavier Morató Arús, Henrik Zetterberg, Kaj Blennow, Nicholas J. Ashton

**Affiliations:** ^1^ University of Gothenburg, Mölndal Sweden; ^2^ University of Brescia, Brescia Italy; ^3^ University of Exeter, Exeter United Kingdom; ^4^ Ace Alzheimer Center, Barcelona Spain; ^5^ ALZpath, Inc., Carlsbad, CA USA; ^6^ Institute for Neurodegenerative Diseases (IND) Florida, Boca Raton, FL USA; ^7^ Lund University, Lund Sweden; ^8^ Network Center for Biomedical Research in Neurodegenerative Diseases (CIBERNED), Madrid Spain; ^9^ University of Gothenburg, Mölndal, Gothenburg Sweden

## Abstract

**Background:**

Phosphorylated tau (p‐tau) is a specific blood biomarker for Alzheimer disease (AD) pathology, with p‐tau217 having the greatest utility. Increased and simplified access to blood biomarkers is crucial for early diagnosis, proper patient management and prompt initiation of disease‐modifying treatments. The DROP‐AD project investigates the capability of finger‐prick collection to accurately measure p‐tau217, neurofilament light (NfL), and glial fibrillary acidic protein (GFAP).

**Method:**

In this prospective study, n=230 participants (mean[SD] age, 71.0 [10] years; 138 females [60.0%]) were recruited from four participating centers. Capillary whole blood was obtained by finger‐prick into three devices (Noviplex^®^; Capitainer^®^ Plasma; Capitainer^®^ B50); not all patients completed all capillary sampling. Capillary cards were shipped to Gothenburg, without temperature control. Samples were extracted by a custom protocol and measured by Simoa. EDTA plasma was collected in all participants; cerebrospinal fluid (CSF) biomarkers were available in 141 individuals. Participants were defined as Aβ positive (Aβ+) using two strategies: a plasma p‐tau217 (ALZpath) >0.63 pg/mL indicating high‐risk^[1]^ and CSF Aβ42/40 <0.063. Statistical analysis included linear regressions, Mann‐Whitney U tests and receiver operating characteristic (ROC) area under the curve (AUC) analyses.

**Result:**

A significant correlation was found for finger‐prick and EDTA plasma for all tested biomarkers (GFAP[n=98], R^2^=0.714; NfL[n=75], R^2^=0.625; p‐tau217[n=73], R^2^=0.561; all P<0.0001). When Aβ+ was defined by plasma p‐tau217, finger‐prick p‐tau217 was increased in Aβ+ (mean[SD], 0.033 pg/mL [0.02]) compared to Aβ‐ patients (0.016 pg/mL [0.03], fold‐change mean[SD], 1.9 [1.5], [n=73], U=175.5, P<0.0001) with high diagnostic accuracy (AUC[95% CI] = 0.867 [0.785‐0.949]). Finger‐prick GFAP was also significantly increased in Aβ+ ([n=35], U=38, P=0.002, AUC=0.838 [0.687‐0.988]). In identifying CSF Aβ+, lower accuracies were observed (p‐tau217[n=58], AUC=0.831 [0.712‐0.949]; GFAP[n=33], AUC=0.714 [0.524‐0.904]) but not statistically different from EDTA plasma. Finger‐prick p‐tau217 and GFAP also associated with cognitive performance (CDR_global_; p‐tau217[n=62], R^2^=0.1011, P=0.0118; GFAP[n=94], R^2^=0.1679, P<0.0001).

**Conclusion:**

This study demonstrates the potential of measuring AD blood biomarkers, including p‐tau217, NfL and GFAP, from finger‐prick collection. We speculate that this simple, self‐executable, temperature‐independent, and reliable method could identify individuals of high risk of AD pathology (p‐tau217) and has potential application for multiple neurodegenerative diseases (NfL and GFAP).

^[1]^Ashton 2024, JAMA Neurol

